# Approaches to Improve EPR-Based Drug Delivery for Cancer Therapy and Diagnosis

**DOI:** 10.3390/jpm13030389

**Published:** 2023-02-23

**Authors:** Md Abdus Subhan, Farzana Parveen, Nina Filipczak, Satya Siva Kishan Yalamarty, Vladimir P. Torchilin

**Affiliations:** 1 Department of Chemistry, ShahJalal University of Science and Technology, Sylhet 3114, Bangladesh; 2 CPBN, Department of Pharmaceutical Sciences, Northeastern University, Boston, MA 02115, USA; 3 Department of Pharmaceutics, Faculty of Pharmacy, The Islamia University of Bahawalpur, Bahawalpur, Punjab 63100, Pakistan; 4 Department of Pharmacy Services, DHQ Hospital Jhang 35200, Primary and Secondary Healthcare Department, Government of Punjab, Lahore, Punjab 54000, Pakistan; 5 Department of Chemical Engineering, Northeastern University, Boston, MA 02115, USA

**Keywords:** EPR-based therapy, passive targeting, heterogeneity, solid tumor, clinical trials, EPR enhancers, nanomedicine

## Abstract

The innovative development of nanomedicine has promised effective treatment options compared to the standard therapeutics for cancer therapy. However, the efficiency of EPR-targeted nanodrugs is not always pleasing as it is strongly prejudiced by the heterogeneity of the enhanced permeability and retention effect (EPR). Targeting the dynamics of the EPR effect and improvement of the therapeutic effects of nanotherapeutics by using EPR enhancers is a vital approach to developing cancer therapy. Inadequate data on the efficacy of EPR in humans hampers the clinical translation of cancer drugs. Molecular targeting, physical amendment, or physiological renovation of the tumor microenvironment (TME) are crucial approaches for improving the EPR effect. Advanced imaging technologies for the visualization of EPR-induced nanomedicine distribution in tumors, and the use of better animal models, are necessary to enhance the EPR effect. This review discusses strategies to enhance EPR effect-based drug delivery approaches for cancer therapy and imaging technologies for the diagnosis of EPR effects. The effort of studying the EPR effect is beneficial, as some of the advanced nanomedicine-based EPR-enhancing approaches are currently undergoing clinical trials, which may be helpful to improve EPR-induced drug delivery and translation to clinics.

## 1. Introduction

Cancer tumors generate an uncommon extracellular matrix, making it challenging for anticancer drugs to infiltrate [1]. In the late 1980s, preferential accumulation of macromolecules into cancer cells was observed. Japanese researchers Hiroshi Maeda and Yasuhiro Matsumura first described the enhanced permeability and retention (EPR) effect in 1986 [2]. This observation was due to the presence of fenestration in damaged tumor blood vessels and poor lymphatic drainage. In hypoxic conditions or inflammation, blood vessels become more permeable. The fast-growing tumor cells in a hypoxic state tend to put in action more blood vessels or submerge existing ones to confront. New blood vessels created due to the process of neovascularization are leaky as they have large fenestrations ranging from 200 to 2000 nm (pore), depending on tumor type, TME (tumor microenvironment), etc. [1]. During the formation of tumors, lymphatic functions are disrupted, resulting in minimal uptake of interstitial fluids, which contributes to the retention of NPs in the tumors as they are not cleared, and stored in the tumor interstitium. This effect is known as the enhanced permeation and retention (EPR) effect. Usually, in normal tissues, the drainage of extracellular fluid into lymphatic vessels often happens at an average flow rate of 0.1 to 2 μm/s, maintaining continuous drainage and renewal [3]. However, when a tumor is formed, the lymphatic function is disrupted resulting in minimal interstitial fluid uptake [1].

The EPR effect is a unique feature of solid tumors related to characteristics of tumors including defective vascular architecture, large space between endothelial cells in blood vessels, abundant vascular mediators, vascular endothelial growth factor, and diminished lymphatic recovery. Therefore, tumor blood vessels are leaky compared to normal blood vessels due to the defective endothelial cells and more vascular permeability, as well as vastly expressed vascular mediators including bradykinin, NO, and VEGF, resulting in selective accumulation of nanodrugs into tumor tissues with little or no distribution in normal cells [4]. Among others, the most prominent pathophysiological factors contributing to EPR-targeted passive tumor targeting include active transcytosis across the blood vessel wall for NPs extravasation and phagocytic uptake by TAMs as a mechanism of NPs retention [2].

In EPR-based passive targeting, TME plays a vital role. One of the crucial metabolic features of fast-proliferating tumor cells is glycolysis, which is the key energy source of cell division and transforms the neighboring milieu into an acidic. This decreased pH of TME can be utilized to deliver pH-responsive NPs to release drugs at a low pH [1,5,6].

The EPR effect is dynamic and a phenomenon of tumor blood vessels, which is mostly dependent on blood flow. Animal models of solid tumors rich in blood flow demonstrated enhanced EPR effects. However, many clinical cancers such as late-stage tumors and refractory tumors are associated with poor blood flow due to the coagulation and thrombi formation exhibiting poor EPR effect [7,8,9,10,11,12]. For such tumors, the improvement of EPR-based drug delivery is necessary by modifying tumor blood vessels, angiogenesis, a vascular edifice, blood flow, etc. The EPR effect can be enhanced by modifying vascular intermediaries such as angiotensin II, nitroglycerin or NO, CO, and enzyme inhibitors [7,8,9,10]. An integration of vascular mediators with a nanodrug may be an important strategy for an enhanced EPR effect. Further, targeting tumor stroma and extracellular matrix, and controlling tumor vessels, may improve EPR-targeted drug delivery to tumors [4].

Tumor blood flow and vascular permeability fluctuate significantly, and blood flow is commonly blocked as the tumor size rises. Early-stage small tumors demonstrate a more uniform EPR effect than advanced large tumors and advanced large tumors reveal heterogeneity in the EPR effect due to the dynamic nature of the EPR effect [13]. It is thus crucial to use EPR enhancers in advanced tumors to improve EPR-based drug delivery. Several strategies may be applied to enhance the EPR effect in solid tumors by modifying tumor blood vessels, angiogenesis, a vascular edifice, blood flow, and other related factors affecting EPR [14]. By controlling tumor vessels using therapeutic nanomaterials as drug delivery carriers, the EPR effect could be increased remarkably. Nanodrugs utilized in anticancer therapy have the benefit of their privileged assemblage in leaky tumors due to the EPR effect [15]. Desmoplastic tumors are allied with dense stromal factors. Therefore, the permeability of the NPs into the tumor is less. These tumors are resistant to NP-based chemotherapy. By modulating tumor blood vessels utilizing nanostructures including NPs, liposomes, micelles, polymers, and nanobiomaterials for drug delivery, the EPR effect may be potentially enhanced for different cancer therapies, such as pancreatic cancer and ulcerative colitis [14,16].

Attempts to engulf the shortcomings of traditional EPR-based nanodrug delivery may include supplementary approaches such as molecular targeting and modification or restoration of the TME [17]. The EPR effect represents several physiological variables, each of which is heterogeneous within a tumor, in a patient, and across patients. As a result, it is necessary to anatomize biophysical factors key to the EPR effect, improve insights into the dynamicity of the EPR effect, fabricate improved nanodrugs effective for passive delivery, recognize patients with high-EPR tumors expected to respond to nanodrugs, improve the imaging methods for detecting and quantifying the EPR in tumors both in animal models and human. Thus, a synergistic combination of multiscale imaging with computational modeling may enhance the knowledge of the EPR effect [18]. Further, modeling to direct the management of the EPR effect, and different features of TME for enhancing nanodrug function may be the crucial approach for the improvement of EPR-based tumor targeting. Integrating multimodal imaging will benefit the quantitative evaluation of the EPR effect in tumors both in human and suitable animal models ensuing advancement of EPR-mediated drug delivery strategies [19].

## 2. Major Advances and Emerging Concepts of EPR-Enhancing Strategies

The strategies to enhance the EPR effect can be broadly classified into pharmacological and physical-based approaches. The pharmacological approaches involve the administration of a drug to interfere with the tumor microenvironment and thereby enhancing the accumulation of nanoparticles at the tumor site. Whereas physical approaches involve the use of an external physical stimulus such as heat or radiation to temporarily improve the permeability in the tumor tissues. These strategies to enhance the EPR are used in both preclinical and clinical settings. In this section, we discuss the recent advancements in EPR-enhancing strategies.

### 2.1. Tumor Vasculature Modulation

**Figure 1:**
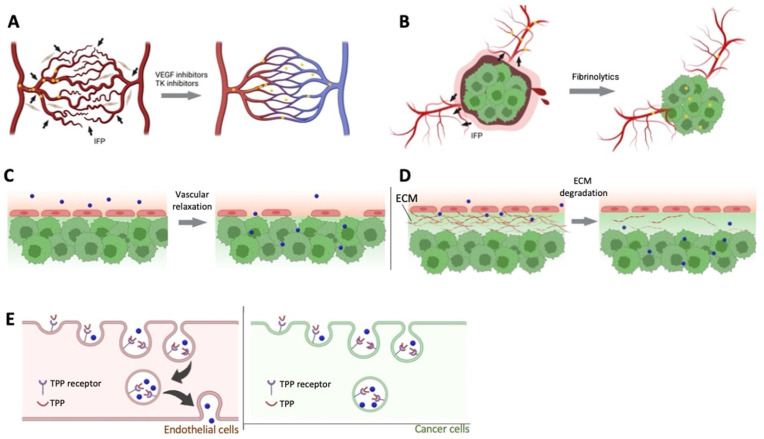
Pharmacological approaches to improve EPR effect. (**A**). Vascular normalizer to improve the blood flow, (**B**). Fibrinolytics co-therapy, (**C**). Enhanced permeability of endothelial cells using vascular mediators, (**D**). Stromal density decreases by ECM degradation, (**E**). Tumor-penetrating peptides enhance transcytosis. Reprinted with permission from [23]. Copyright (2023), with permission from Elsevier.

In clinical settings, one of the main challenges for the delivery of macromolecules and nanomedicine to the cancer site is due to the heterogeneity of the EPR effect in solid tumors [20]. Several factors result in the heterogeneity of the tumor such as stage and size of the tumor, primary metastatic nature, and pathological characteristics. Two main approaches are employed to restore effective blood perfusion to the tumor site. This results in the mitigation of tumor heterogeneity and vasculature, normalizing disorganized tumor vasculature, and unblocking the occluded vessels are the two main approaches in achieving uniform drug delivery to the tumor site as shown in Figure 1 [13,21,22]. 

### 2.2. Normalization of Vasculature

One of the classical approaches is to administer antiangiogenic compounds and deprive the tumor tissue of oxygen and nutrients. In clinical settings, this approach has been proven to be ineffective when used alone [23]. Recently, research groups such as Jain, et al., have used the combination therapy of angiogenesis inhibitors along with chemotherapy [24], where the angiogenesis modulators are used to normalize the vasculature and improve the EPR effect–mediated drug delivery and efficacy of the cancer treatment. An intermediate dose of anti-VEGF receptor two antibodies (DC101) was used to successfully normalize the blood vasculature in and around the tumor site, thereby reducing the necrotic and hypoxic regions [25]. Further, the research group administered a combination of Doxil (around 125 nm in size) or Abaxane (around 12 nm in size) in breast tumor-bearing mice and observed a significant (around threefold) accumulation of smaller nanoparticles [26]. This concludes the DC101 improved tumor vascular normalization leading to a smaller and homogenous pore on the tumor vessel wall [25]. Another class of drugs used to aid in vascular normalization is tyrosine kinase inhibitors (TKIs), a small molecule class of drugs. Several research groups employed treatment with erlotinib, an EPR enhancer which is a TKI against epidermal growth factor receptor (EGFR). This improved the accumulation of human serum albumin-bound paclitaxel (HAS-PTX) in vivo in tumor-bearing mice such as head and neck carcinoma cells (SCC7), colon carcinoma cells (CT26), and breast cancer cells (4T1) [27]. A significant tumor size reduction was observed in models with combination therapy of erlotinib and has-PTX in comparison to the HAS-PTX group alone. A higher vascular normalization resulted in a higher distribution of hasHSA-PTX to the tumor site. Another TKI, Imatinib, inhibits VEGF-independent angiogenesis and also improves the delivery of nanoparticles to the tumor site, which was observed in A459 xenograft tumor models (lung carcinoma) [28]. Most research groups have reported that vascular normalization results in pore size reduction in the tumor vasculature. One of the problems with vascular normalization is the accumulation and quick clearance of small-sized nanoparticles. Research groups such as Xiao et al. used a combination of cediranib (VEGFR TKI) and enzyme-responsive size changeable gold nanoparticles (from around 40 nm to 300 nm upon enzymatic activity) [29]. The size-changeable gold nanoparticles have shown an around twofold increase in the tumor size suppression in the subcutaneous 4T1 tumor model than the fixed size nanoparticles. The increase in size led to increased residence time in the tumor tissue and improved the antitumor effect of size-changeable gold nanoparticles that increased with the coadministration of cediranib. In addition, celecoxib a cyclooxygenase-2 (COX-2) inhibitors also act as an angiogenesis inhibitor and result in vascular normalization and improved EPR effect in the tumor site [30]. The choice of therapy for vascular normalization must be chosen wisely since this approach is effective in delivering small-sized nanomedicines in comparison to the larger ones.

### 2.3. Fibrinolytic Co-Therapy

Cancer mortality and morbidity are contributed to by a hypercoagulative state of malignancy in patients [31]. Vascular occlusion in tumors can be caused due to several factors such as tissue factors that secrete proinflammatory cytokines being overexpressed by tumor cells, platelet activation due to cytokine-activated endothelial cells, and leukocytes overexpressing tissue factors. All these factors result not only in vascular occlusion but also tumor heterogeneity of the EPR effect and thereby resulting in poor drug delivery. Fibrinolytic therapy helps in dissolving fibrins in occluded vessels and improving the tumor vasculature [32,33,34,35,36]. Research groups such as Zhang et al. preadministered tissue plasminogen activator (tPA) with paclitaxel-loaded nanoparticles to de-compress the tumor vessels in A549 tumor xenograft mice. The tPA is a thrombolytic drug that degrades fibrins, and the pretreatment decreased the number of fibrins at the tumor vessel walls. The pretreatment group showed a higher accumulation and enhanced penetration depth of 115 nm nanoparticles at the tumor site in comparison to the saline group [37]. In another study, researchers reported that pretreatment with tPA has not affected biodistribution. However, improved the penetration of Doxil^®^ into B16F10 tumors [38]. Although co-therapy with fibrinolytic has shown promise and great potential in increasing the EPR effect in delivering nanomedicine, tPA’s low half-life and specificity remain a challenge.

### 2.4. Bradykinin Mediators and Bradykinin

Activation of bradykinin receptors B1 and B2 results in increased vasodilation, disruption of endothelial adherens junctions, and actomyosin cytoskeletal contraction of endothelial cells [39]. Several tumors overexpress kinin receptors resulting in higher permeability of tumor vasculature. Since the half-life of bradykinin is short lived and it has the risk of pain induction, some research groups use drugs that inhibit angiotensin-converting enzyme (ACE). These ACE inhibitors (ACEi) inhibit the conversion of angiotensin I to angiotensin II and thereby inhibiting the degradation of bradykinin. ACEi drugs such as enalapril are used to improve the permeability of the vasculature further and improve the accumulation of nanomedicine at the tumor site. Some research groups have used captopril which is a more potent ACEi than enalapril. Captopril usage has improved the accumulation of paclitaxel-loaded nanoparticles in U87 glioma xenograft tumors [40,41,42].

## 3. Approaches to Improve the EPR Effect in the Tumor Microenvironment

**Figure 2:**
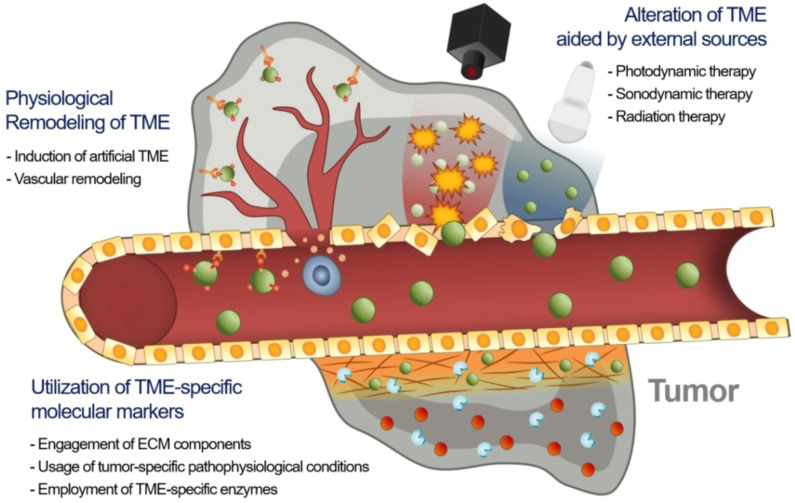
Strategies to enhance EPR-based drug delivery targeting the tumor microenvironment. Adapted with permission from [17].

The therapeutic efficacy of nanodrugs is affected by the heterogeneity of the extent of the EPR effect in tumors, which causes disappointing therapeutic results in animal models and humans. As a result, novel approaches are required to advance EPR-based drug delivery in tumors [17]. There may be three major approaches to improve the EPR effect in TME, namely utilizing specific molecular markers of TME including extracellular matrix (ECM) constituents, tumor-specified pathophysiologic settings, and TME-specified enzymes with nanodrugs; use of external physical methods for remodeling TME; and physiological modification of TME by inducing artificial TME or promoting vascular renovation (Figure 2).

Accessorizing nanodrugs with ligands that can target TME is one of the best effective ways to improve the targeting ability of nanodrugs for overcoming tumor heterogeneity and enhancing the EPR effect. The EPR-based drug delivery is allied with the ECM heterogeneity and physiologic sceneries in the tumors [43]. The likely molecular targets in ECM include ECM proteins (collagen, fibronectin), proteoglycan, growth factor receptors, and transmembrane receptors [44]. Therefore, disintegrating these protein molecules by targeting slacken, the ECM mediates the delivery of nanodrugs into the tumor sites.

Integrin is a tumor-specific marker of angiogenic function in ECM among the transmembrane factors [45]. Playing a crucial function in tumor proliferation and metastasis is αvβ3 integrin, which is overexpressed on angiogenic vessels. Thus, targeting αvβ3 enhances the delivery of NPs to the angiogenic vessels, improving the extravasation of nanodrugs in tumors [46]. NIR fluorescent NPs congregated with cyclo[-[D-Ala-L-Glu-D-Ala-L-Trp)_2_] were altered with RGD (Arg-Gly-Asp) moieties and then loaded with the chemo drug, epirubicin. The nanoassembly demonstrated enhanced accumulation in tumors through αvβ3 binding and the EPR effect, causing improved therapeutic efficiency [47]. The self-assembled nanosystem altered with RGD did not show any cardiotoxicity. RGD-based drug delivery to tumor ECM has shown promise. However, the RGD-based peptide delivery system may be allied with severe immune activation [48]. The undesirable immune stimulation due to the integration of RGD on NPs may be reduced by loading cytotoxic drugs in the nanosystem. For example, encapsulation of the cytotoxic drug doxorubicin into RGD-based nanodrugs targeting ECM knocks off fatal reactions and immunotoxicity, inhibiting the inadvertent lethal IgG response in patients. This study indicated that precise regulation over ECM-targeted peptide-based NPs reduces immunotoxicity, and its side effects, and enhances drug delivery in addition to EPR, as well as the therapeutic effects [48].

Overexpression of EGFR is detected in different cancer cells, which is involved in several molecular and cellular pathways including apoptosis inhibition [49]. Mostly utilized to target EGFR on tumor cells are mAbs, such as cetuximab or their fragments. EGF ligand is promising for targeting EGFR in tumors [50]. To target EGFR, liposomes attached to EGF have been developed for oxaliplatin delivery into tumors. EGF-liposome conjugates remarkably reduced the IC_50_ value of oxaliplatin in colorectal cancer cells which overexpressed ERFR, though not in the EGFR-negative colorectal cancer cells [51]. This study indicated that the enhanced EPR effect and therapeutic efficiency may be achieved by targeting EGFR in TME.

HA (hyaluronic acid)-based nanodrugs could be assembled more at the tumor by EPR effect through binding to CD44 [52]. A thermo-responsive self-assembled HA-based nanosystem for codelivery of MMP (matrix metalloproteinase) inhibitor (marimastat) and paclitaxel conjugated with HA was fabricated for duple targeting of TME and tumor cells. The developed nanoplatform fostered the accumulation of drugs into tumors, demonstrating tumor inhibition, and metastasis inhibition. The study showed that the integration of TME-targeting with HA-based nanodrugs with a TME regulator is a prospective approach for cancer therapy [53].

Further, HA-based CPP (cell-penetrating peptide)-modified LNPs were developed to enhance the EPR-based drug delivery into hepatocellular carcinoma. The HA-based CPP-modified LNPs effectively infiltrated into the ECM and assembled into the tumor by enhanced EPR effect, which was detected by the imaging technique.

Dual targeting approaches with EGFR and CD44 are also effective for enhancing EPR-based drug delivery, which may decrease the uncertainty of a single targeting strategy [54,55]. PEGylated recombinant human hyaluronidase, PEGPH20, has exhibited improved therapeutic possessions in vivo, hindering tumor growth and metastasis progression. HA-degradable PEGPH20 in integration with gemcitabine has demonstrated enhanced therapeutic effects in animal models [56,57].

Hypoxia (oxygen-deficient environment) is the consequence of the high demand for nutrients and oxygen in malignant tumors [58]. Hypoxia is allied with tumor angiogenesis and metastasis and may lead to MDR in tumors [59]. Nanodrugs can be fabricated to bind hypoxia-specific molecular markers and silence the gene expression through siRNA therapy or by delivering cytotoxic agents to hypoxic tumors. Phosphatidylserine is a phenomenon usually allied with apoptosis. A liposomal protein, Saposin C bonded to phosphatidylserine, may be used to fabricate NPs to bind to hypoxic TME [60,61].

Another TME-targeted effective approach may be the delivery of the siRNA targeting hypoxia-related gene, *HIF-1α*, to treat hypoxic tumors. The *HIF-1α* gene is translated into the HIF-1α protein, which is allied with the stimulation of a series of genes that may worsen the tumor state [62]. A cationic micellar NP combines HIF-1α siRNA and the NP-siHIF nanosystem that effectively reduced tumor cell proliferation, angiogenesis, and migration, and hindered tumor growth as well as reduced MDR1 expression both in vitro and in vivo [63,64].

A hypoxia-responsive copolymer system was developed for siRNA delivery to tumors [65]. A lipid-conjugated PEG-shielded PEI-siRNA nanoplatform was fabricated for delivery of siRNA to hypoxic TME. Herein, the azobenzene linker degraded to release the PEG layer revealing the siRNA into hypoxic TME and permitting its hypoxia-dependent uptake into A549 lung tumor cells in a spheroid model. The nanoplatform, which is hypoxia sensitive and bearing DOX, deconstructed and released DOX in a hypoxic environment. The nanodrugs exhibited effective accumulation into a tumor and inhibited tumor growth upon systemic delivery. Thus, hypoxia-sensitive nanodrugs permit systemic synergistic effects in tumors.

An incipient TME-targeting approach is the utilization of an acidic environment in TME fabricating tumor-specific nanodrugs. The acidity of TME is a significant therapeutic target. The acidic extracellular TME could reverse the surface charge of the nanodrugs, leading to NP accumulation in the TME. The pH-responsive NPs could improve the privileged EPR effect and aggregation of the nanodrugs in tumors [66,67,68].

Further, modification of pH in TME by NPs and suitable chemical mediators may enhance the uptake and treatment effect of nanodrugs to improve the therapy result. Liposomal DOX and NaHCO_3_ demonstrated a 21-fold enhanced drug uptake compared to the reference in a 24 h post injection in a mouse model of TNBC tumors [69]. Thus, alteration of the pH of TME embraces the potential for promoting TME towards enhanced therapeutic function.

TME-specific enzymes may be utilized to activate drugs in drug delivery systems for enhanced EPR-based drug delivery. This strategy has several benefits, such as enhanced tumor aggregation, reduced systemic toxicity, and improved therapeutic effects. A prodrug consisting of peptide and DOX self-assembled to form NPs and was aggregated into the tumor by the EPR effect. It was then activated by cathepsin B, which is overexpressed in tumors, by releasing free DOX in tumors [70]. Therefore, a peptide-based self-assembly approach would be prospective for enhancing the EPR effect.

Several enzymes, such as MMP and caspase, have been applied to enhance the EPR-based drug delivery of nanodrugs [69,71]. The apoptosis induction is primarily necessary to originate the overexpression of caspase-3 in tumors in the case of caspase-3-responsive prodrugs [72]. The stimulation of caspase-3 and triggering prodrug-induced apoptosis and improved therapy efficiency. The Onivyde drug is based on irinotecan, and it has demonstrated enhanced response and therapeutic efficiency in clinical use for cancer therapy [72,73]. Liposomal irinotecan, in combination with radiation therapy (RT), inhibits cancer cells in apoptotic conditions, however, not irinotecan itself. Liposomal irinotecan can assemble in TME, and be metabolized to an active form by TAMs.

Nanodrugs solely dependent on EPR effect are less effective due to the poor accumulation of tumors. However, combination therapy of nanodrugs and PDT can inhibit resistant cancer cells by improving the tumor infiltration of the nanodrugs [74]. Nano-photosensitizers, isophthalic acid/layered double hydroxide, and hybrid nanosystems exhibited enhanced cytotoxicity with significantly a low IC_50_ value and safety profile, which was allied with a higher EPR effect [59].

Sound dynamic therapy is a promising cancer therapy. Sonosensitizers are prompted by ultrasound to form ROS for inhibiting cancer cells in tumors. A combination of SDT with nanodrugs is a prospective approach for cancer therapy. SDT has a adequate deep tissue penetration ability than light in PDT, which is desirable for the treatment of deep-seated tumors and enhancement of the EPR effect of nanodrugs. Sonosensitizer-containing NPs were developed to enable transferrin-facilitated endocytosis for deep tissue infiltration [75]. The developed NPs released sonosensitizers protoporphyrin IX by transferrin intervention to overcome the tissue barrier and enhance the EPR effect in tumors. Further, high-intensity focused ultrasound may be utilized to improve the EPR effect of nanodrugs in tumors [76].

NPs with RGD, that target TME, in integration with RT, have demonstrated that the radiation can influence endothelial cells and blood vessels and enhance the EPR effect as well as the therapeutic response to chemotherapy [77].

The prodigious advancement of NPs has markedly expanded the utilization of PDT, SDT, and RT in integration with nanodrugs for the enhancement of EPR-based drug delivery and therapeutic efficacy in cancer treatment. Thus, the enhancement of the EPR effect by PDT, SDT, and RT with the nanodrugs has synergistically improved anticancer therapy [78].

Physiological renovation of TME significantly enhances the delivery of nanodrugs into the tumors. Remodeling of TME focused on the biological modification of the structural characteristics of tumors and their environment to improve nanodrug delivery to the target tumor cells [44,79]. An artificial azide reporter emerging from the metabolic precursor (Ac_4_ManNAz) can be introduced on the tumor cell surface [80,81]. This strategy presented artificial receptors on the surface of tumor cells, irrespective of tumor type as a substitute for using natural receptors. The strategy exhibited a way to overwhelm the heterogeneity of intensity of the EPR effect and to enhance the EPR-based drug delivery [82].

Further, artificial ECM may also be built utilizing laminin protein-mimic peptide-based NPs [83]. Laminin-mimic peptide-based NPs aggregated in the tumor through the EPR effect and relocated into ECM, which subdued lung metastasis in breast tumor and melanoma models.

Palladium encapsulated in NPs exhibited effective catalytic activity in animal models in vivo [84]. This alteration of catalytic activity in tumors in vivo may be considered as generating artificial TME for improved drug delivery. Herein, NP-palladium aggregated in tumors by EPR effect and stimulated a DOX prodrug in a tumor.

TGF-β plays a crucial role in TME renovation, controlling angiogenesis, and preventing the expansion of the T cells [85,86]. Thus, remodeling of TME through the inhibition of TGF-β permits NPs to infiltrate into targeted tumor tissues significantly. A low dose of TGF-β inhibitor effectively modified TME, tumor vasculature, and enhanced the EPR effect [87].

The delivery of EPR-dependent nanodrugs potentially depends on the properties of tumor vasculature, which could affect the EPR effect. As a result, vasculature remodeling utilizing a drug delivery system has been a prospective approach [88,89].

Tumor vessel alteration could be an efficient approach to impact the intensity of the EPR effect. Remodeling of tumor vasculature utilizing angiogenic mediators or external stimuli modulates the function of the vessels affecting infiltration and disruption [90].

Further, strategies that increase the endothelial pores which assist in the entry of nanodrugs to extravasate into TME by utilizing NO, nitroglycerin, TNF-α, angiotensin, etc. are prospective for EPR enhancement and improvement of therapeutic effect. For example, NO-releasing NPs integrating a photosensitizer (IR780) and chemotherapeutic paclitaxel remarkably reduced tumor growth by enhancing tumor vascular permeability and leading to the inhibition of tumors [91,92]. Further, the regulation of tumor blood vessels in TME may be attained by the delivery of anti-VEGF antibodies or VEGF receptor inhibitors [93,94].

Finally, the restrictions associated with EPR-based drug delivery of nanodrugs may be overcome by integrating with accompanying approaches, such as further molecular targeting, physical modulation, or physiological alteration of the TME to enhance the EPR effect and improve therapeutic efficacy.

Mild hyperthermia in the range of 39–42 °C is another important strategy to enhance the EPR effect that is achieved by heating tumors or high-intensity focused ultrasound. After the discovery of “ThermoDox”, various research groups have conducted trials to improve the delivery of cancer chemotherapeutics by preparing temperature-sensitive formulations [95,96,97,98].

The use of ultrasound waves is more beneficial to induce sonoporation within the cell membranes of deep tumors that increase vascular permeability. The combined effects of microbubbles and focused ultrasound to enhance EPR-mediated drug delivery have been investigated in various preclinical and clinical trials [99,100,101].

## 4. Dynamics of the EPR Effect and Strategies to Improve the Therapeutic Effects of Nanomedicines by Using EPR Effect Enhancers

### 4.1. Dynamics and Heterogeneity of Vascular Structures in Human Tumors

The theoretical expectations of the EPR effect implicated in animal models have been considered a major guidance phenomenon for the clinical translation of cancer therapeutics. However, the clinical outcomes of this phenomenon highlighted the poor manifestation of this central paradigm. Human solid tumors are heterogeneous as compared to animal and laboratory models in terms of the permeability of blood vessels, the density of the extracellular matrix, and other dimensions (Table 1). The heterogeneity of this complex phenomenon has also been evaluated among different persons and tumors at different sites of the same person during clinical investigations. Therefore, there is a need to step ahead of the concept of “one-size-fits-all” to personalize the treatments based on specific oncology cases.

Among various critical features, the invasive nature of cancer tissues and rapid growth creates solid stress on angiogenic blood vessels that infiltrates diverse cells. The hypoxic environment is created in response to the production of angiogenesis-enhancing factors. These factors known as vascular endothelial growth factors, VEGF are responsible for the survival of endothelial cells and the leakiness of vessels. The sluggish flow of blood is contributed to by the impairment of vascular perfusion and large lumen diameter. All these factors, along with the malfunctioning lymphatic drainage system, attenuate the nanocarriers to accumulate in the early stages of small tumors.

Tumors in humans are poorly diagnosed in the initial stages, which is mainly responsible for the limiting effects of EPR after drug release [102]. The blood supply is occluded due to the formation of thrombi and clots in the advanced stages of cancer, which is responsible for impaired drug delivery in deep tumor tissues.

As tumors grow, the coagulation system is also activated, which is responsible for the poor accumulation of drugs due to avascular areas and embolization of tumor tissues [32]. The poor lymphatic system of rapidly growing tumors, furthermore, creates an osmotic pressure that will enhance the retention of therapeutics. The other mechanical pressures and physical barriers due to the rapid growth of tumor mass may also contribute to the heterogeneity of the EPR effect and declined efficiency of nanotherapeutics in human tumors [103]. Therefore, there is a strong research desire to enhance the EPR effects by different pharmacological and physical means.

#### 4.1.1. Pharmacological Strategies to Improve the EPR Effect

Inflammatory mediators like kinin and bradykinin facilitate the vascular permeability of deep-seating tumors via an opening of endothelial cellular gaps [104]. Angiotensin, being similar to kinin in pharmacological effects perhaps due to a similar sequence of amino acids at the C-terminal, is also responsible for the EPR effects-based tumor accumulation of drugs.

The renin–angiotensin system (RAS) and kallikrein–kinin systems (KKS) mediate inflammation, thrombosis, and increased vascular permeability when activated through a series of proteolytic enzymatic degradations. RAS is activated when nitric oxides (NO), prostaglandins, or low sodium levels contribute to the secretions of renin that cleave angiotensinogen to angiotensin I. These reactions mediated by angiotensin-converting enzymes (ACE-I and ACE-II) play a critical role in the regulation of blood pressure, inflammation, vascular permeation, coagulation, and fibrosis [105]. The inhibitory effects of this enzyme mediate the release of bradykinins, a major player of KKS through kinin receptors [106]. The degradation of bradykinins is responsible for low EPR effects. The pharmacological approaches to increase the levels of bradykinins are favorable for the enhancement of EPR effects.

The plasma and tissue KKS are responsible for the synthesis of short-lived kinins that are transformed by proteolytic cleavage into biologically active bradykinins. The activation of plasma and tissue kallikreins by factor X II, also known as an important initiator of blood coagulation and aminopeptidases, respectively, results in the synthesis of bradykinins [107].

The pharmacological strategies to improve the EPR effects involve the use of pharmacological agents that exert vaso-modulatory effects like that of bradykinins. The use of ACE inhibitors, NO, carbon monoxide, and CO-producing agents are common strategies to improve the EPR effects by improving nanomedicine accumulation and vasodilatory actions of these EPR enhancers (Table 2).

#### 4.1.2. Physical Strategies to Improve the EPR Effect

Various research groups have contributed to the EPR effect enhancement strategies by employing physical means such as mild hyperthermia, photodynamic activation of tissues, radiation therapy, and ultrasounds [90,113,114,115,116,117,118,119].

Photodynamic therapy is one of the most important physical modalities to improve the therapeutic effects of nanoparticles by physical means. This involves the production of reactive oxygen species, ROS, after laser irradiation that kills tumor cells. However, the hypoxic conditions in the tumor microenvironment and improper blood supply to the tumor mass present as a major barrier in the clinical progress of this physical therapy [118].

Ionizing radiations can reduce the cellular density of tumors by the liberation of cytotoxic radicals and regulate vascular endothelial and fibroblast growth factor expression. The combined effect of radiation therapy decreases the fluid pressure and facilitates the improved accumulation of tumor-targeted nanotherapeutics. Liu and coworkers prepared polymeric micelles and utilized radiations as an external trigger to release the drugs (doxorubicin, docetaxel, and paclitaxel) from micelles. Enhanced release of doxorubicin was observed in the presence of photosensitizer chlorin (Ce6) and radiation exposure [120].

## 5. Applications of Advanced Imaging Technologies for Visualization and Quantification of EPR-Induced Nanomedicine Distribution in Tumors

Imaging is significant for tumor targeting and the development of nanodrugs for cancer therapy since it helps to represent the effectiveness of tumor targeting and heterogeneity of the EPR effect [121]. Molecular imaging modalities have the potential for detecting EPR effects to formulate better therapeutic NPs and select patients with a high EPR effect [122]. Three major groups of imaging techniques are utilized in therapeutic NP research. These are noninvasive imaging techniques such as nuclear imaging (PET, SPECT), MRI, CT, and US; optical imaging; and mass spectrometry imaging. Each imaging technique affords imaging evidence at dissimilar measures with its own merits and demerits. Thus, selecting astutely and integrating multiple techniques will deliver sufficient information on therapeutic NPs distribution in tumors [19].

A huge disparity in the EPR effect exists between tumor types, sizes, and sites, mostly associated with inconsistency in the construction and function of tumor blood vessels [123]. The differences in the composition of TME may lead to intertumoral heterogeneity in nanodrug aggregation and therapy outcome [124]. Further, preclinical animal models of tumors usually overrate the EPR effect compared to tumors in humans, which obscures the clinical advancement of nanodrugs [125].

Image-guided system pharmacology of anticancer nanodrugs is of importance for the alteration of TME and the enhancement of the EPR effect in tumors. Comprehensive studies have focused on integrated multiscale imaging with computational models to navigate intuitions about the EPR effect and utilized modeling to operate the handling of the EPR effect and TME to develop and improve nanodrug therapy. A harmonious integration of imaging with system-level computational approaches for the clinical development of nanodrugs has been growing as technologies are improving for increased resolution, multiplexing, and examining the heterogeneity at the single-cell level [18].

Imaging assists in determining nanodrug distribution, target tumor site accumulation, and drug release [126]. Imaging enables patient lamination via different approaches including companion nanodiagnostics, nanotheranostic, conventional imaging techniques, and immunohistochemistry. Multiscale imaging modalities could analyze the heterogeneity of the nanodrugs’ spatial distribution in the tumors [19]. The microcomputed tomography (μ-CT) imaging approach was established to image the intratumoral distribution of contrast agent-loaded PEGylated liposomes. This semiquantitative technique was utilized to determine whole 3D liposome distribution in tumors with 17 μm resolution in different PDX models. Further, high-resolution ex vivo μ-CT was utilized to study the spatial distribution of drugs in different tumor models [127]. This study identified vessel spread and vessel support as significant factors for effective liposome aggregation in tumors.

HA-conjugated fluorescent dyes were used to image pancreatic tumors. The results exhibited that the molecular weight of HA and the properties of conjugated dyes influence the imaging outcome [128]. Further, star polymers chelated with MRI contrast agents and radioisotopes were used for imaging and radiotherapy of cancerous lesions by manipulating EPR-based aggregation in the tumors [121].

The combination of nanodrugs with imaging modalities is crucial for visualizing tumor-targeted drug delivery processes, and inter- and intraindividual heterogeneity in EPR-based drug delivery [126]. In preclinical studies, integration of imaging with a nanodrug delivery investigation allowed quantifiable evaluation of nanocarrier biodistribution, target area aggregation of nanodrugs, and enhanced drug release. In a clinical setup, imaging has been evolving as an aid for the lamination required for the translation of anticancer nanodrugs.

For visualization and quantification of nanodrug circulation behavior, biodistribution, and target tumor aggregation, imaging agents have been encapsulated within or conjugated to NPs and imaging them utilizing different imaging techniques [129]. For this purpose, existing imaging techniques such as optical imaging, radionucleotide imaging, and newer imaging modalities are applied to display nanodrug biodistribution and tumor site aggregation.

In addition to MRI, optical imaging approaches, and fluorescence techniques, FRET has also been utilized for observing drug release [130,131]. Cy5.5-PLGA NPs and an encapsulated Cy7X model drug was utilized to investigate the NP–drug association and release kinetics with in vivo optical imaging in a mouse model of the TNBC cell line MDA-MB-231. Upon intravenous (i.v.) injection, the FRET/Cy5.5 intensity ratio was determined to evaluate NP-drug association demonstrating Cy7-X with the polymeric matrix had the highest NP association and inhibited early drug release [126].

Therapy heterogeneity is crucial for drug resistance. Heterogeneous drug distribution happens for drugs of almost all sizes, and therapy shows no desired effect [131,132]. Further, undesired aggregation of drugs into healthy cells may cause toxicity [131]. Further, therapy heterogeneity may produce discrete microenvironments within tumors resulting in intra- and intertumoral heterogeneity, affecting clinical results.

Furthermore, to achieve optimum biodistribution and tumor accumulation, it is also important to release therapeutic payload from nanocarriers to tumors. For noninvasive quantification and visualization of drug release, nanotheranostic has been developed through the coentrapment of chemotherapeutics and imaging agents into the delivery platform. In this case, the imaging agent must furnish an unlike signal when it is bound into the nanocarrier versus when it is free upon release. As a result, PET and SPECT-based probes are less suitable for imaging drug release and optical and MRI-based contrast agents hold landscapes better for this purpose [130].

CT imaging for nanodrugs can detect tumor transport properties. However, dynamic contrast-enhanced (DCE)CT has been utilized to evaluate the intratumoral perfusion, permeability, and aggregation of CT contrast agent-carrying NPs in tumors in mice [19,132,133]. Intratumoral perfusion is allied with liposome accumulation. Thus, DCE-CT is beneficial for identifying patients expected to respond to liposomal drug therapy [134].

Usually, macroscopic optical imaging techniques are utilized in preclinical animal imaging as a substitute for full-body imaging techniques such as PET/SPECT, MRI, and CT. BLI is used to assess nanodrug efficiency based on the endogenic luminescence of tumors to confirm NPs diagnostics or theranostic prospects and to integrate with other imaging modalities [19,134,135,136].

The incorporation of MRI contrast agents or optical imaging agents into nanocarriers for the noninvasive evaluation of drug release in vivo, in real time, is a promising strategy. This approach will offer a wealth of information in drug release kinetics in vivo. Further, imaging of tumor blood vessel density, perfusion, permeability, and the composition of tumor ECM are crucial for achieving prospects in anticancer nanodrug development. To improve the clinical result of nanodrugs, astute approaches, and pragmatic translational circumstances are essential for the EPR-based tumor targeting and therapy [126]. Integration of a theranostic may contribute to enhancing EPR-targeted drug delivery and treatment of tumors.

The combination of imaging data collected from multimodal hybrid methods provides a prospect to integrate information related to NP distribution and TME on multiple scales [19]. This approach offers synergistic advantages over the use of a single modality. In general, imaging techniques in clinics integrate to unite functional (SPECT/PET) with anatomical (CT and MRI) information obtained on the same scale. Different types of NPs for multimodal imaging have been established such as modified Gd_2_O_3_ NPs, gold NPs, ^64^CuInS/ZnS QDs, Bi_2_S_3_ NPs, and liposomal nanocarrier conjugated ^99m^Tc [137,138]. On tissue and cellular scale, imaging combination of optical imaging and mass spectrometry imaging techniques have demonstrated promise [139]. Fluorescence MSI has been integrated to depict local drug release and record unlabeled therapeutic drug circulation, which is offered to track the drug metabolites [140]. The hybridization of MSI and imaging mass cytometry (IMC) with immunohistochemistry and fluorescence in situ (hybridization) allowed the monitoring of drug levels within tumor regions [19].

For achieving multiscale imaging evidence, optical imaging and MSI have been harmonized with clinical imaging [141]. Utilizing a duple of fluorescent and MRI agents, the high sensitivity of the fluorescent was accompanied by the MRI’s aptitude for deep-tissue imaging and high spatial and temporal resolution [142,143].

By integration of molecular imaging and bioanalytical techniques, MSI and IMC determined the biodistribution of drug and drug metabolites delivered via PLGA-PEG NPs and overlaid this with imaging of AZD2811 in the context of TME markers in different PDX animal models. By staining the multiple biomarkers of the TME on the same tumor section using IMC, and coregistering and combining data from both imaging techniques, features in the region with the highest nanodrug distribution were determined. The delivered nanodrug was associated with tumor sites higher in macrophages, as well as high stromal regions [19,144].

Combining 2D MSI and optical imaging with 3D images generated by SPECT, PET, CT, and MRI is challenging. Integration of 2D MSI with 3D MRI was able to monitor MRI contrast agents [145]. Utilizing distortion electrospray ionization mass spectrometry (DESI-MSI) to monitor Gadoteritol for characterizing intratumoral heterogeneity was promising. Herein, contrast agent mass spectrometry imaging (CA-MSI) was developed using MRI contrast agents ([Gadoteridol+K]^+^ and ([Gadoteridol+Na]^+^) targeted to the tumor, as a label, which revealed tumor heterogeneity in absence of a mass profile. Breast cancer tumors developed in mice were investigated by CA-MSI using Gadoteridol, revealing tumor margins and vasculature by localizing the contrast agents. Thus, a combination of MSI with other techniques is prospective for bridging the gaps between unalike scales [145,146]. Multiscale and multiaspect imaging for NPs, drugs, and tumor environment, is a prospective approach to improve the characterization of a spatially heterogenous effect and greatly contributes to highly effective nanodrug development for efficient cancer therapy.

**Figure 3:**
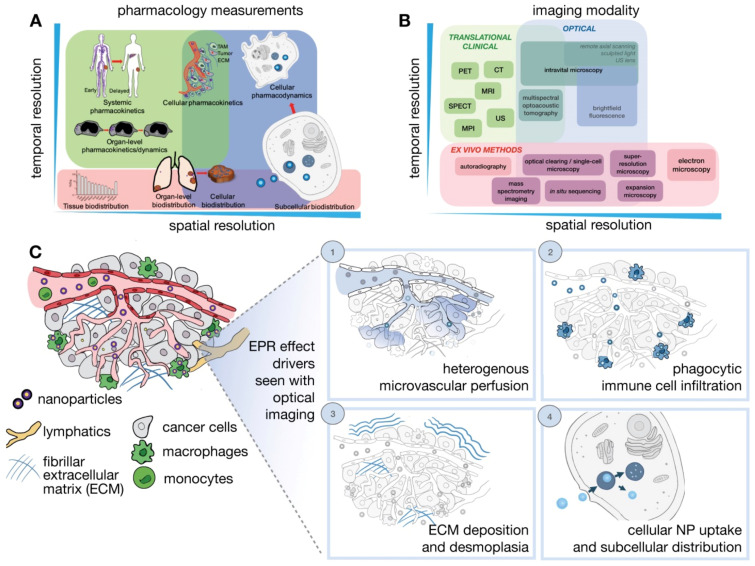
Imaging NP pharmacology and the EPR effect across multiple spatial and temporal scales. (**A**) Pharmacokinetics and pharmacodynamics (PK/PD) of NPs (**B**). Imaging strategies and (**C**) IVM modalities are particularly suitable for imagining dynamic and microscopic courses of the EPR effect and the TME. Adapted with permission from [18].

Determinants of the EPR effect and nanodrug performance happen across manifold spatial and temporal scales ranging from systemic biodistribution of nanodrugs to their delivery and consequences upon distinct cells (Figure 3) [18]. However, a single imaging modality cannot fully harbor the different levels of valuation required. As a result, multiple imaging modalities are usually combined to offer a complementary and comprehensive outlook. Generally, in vivo imaging approaches needed compromise between depth, resolution, and image contrast. In quantitative imaging technology, the imaging signal is better and can be utilized for full-body imaging. These modalities include the laboratory-based technique CLSM (confocal scanning microscopy), and clinically relevant imaging techniques such as PET, and MRI. Several new imaging modalities are being developed such as light sheet microscopy, remote axial scanning, ultrasound lenses, optoacoustic lens imaging, magnetic particle imaging, etc. These new modalities will likely extend possibilities for the imaging of nanodrugs [147,148].

Cancer nanodrugs rely on the EPR effect for effective accumulation into the target site [2]. However, the EPR effect is very heterogeneous, and its level is dynamically varied through the progression of therapy. An effort has been made to longitudinally study the dynamics of the EPR effect upon single- and double-dose nanotherapies with fluorophore-labeled and paclitaxel-loaded polymeric micelles using CT-fluorescence molecular tomography imaging. It was observed that double-dose nanotaxane therapy interindividual heterogeneity in EPR-based tumor aggregation enhances the therapy. Further, for dual dose treatment, tumor aggregation was enhanced over time, from 7% injected dose per gram upon the first administration to 15% injected dose per gram upon the fifth administration, contributing to efficient tumor growth inhibition. Thus, during nanodrug therapy evaluating the dynamics of EPR-based drug delivery using imaging is crucial for therapy prediction and clinical translation. Cy7-labeled and paclitaxel-loaded micelles were fabricated to longitudinally represent and enumerate nanomedicine tumor aggregation during nanotaxane therapy of 4T1 murine TNBC tumors. The result demonstrated aggregation of micelles in tumors. Heterogeneity in EPR-based tumor targeting was detected during nanodrug therapy. Disproportionally more tumor accumulation was observed for double dose than single dose with an enhanced therapeutic effect. Thus, imaging tumor accumulation multiple times during therapy may be suitable for predicting therapy outcomes instead of imaging once at the beginning of treatment [2].

Nanoparticle behavior in vivo is very complex. As a result, a profound knowledge of in vivo behavior, targeting mechanism, and nanoparticle’s explicit engagement with cell populations such as tumor, stromal, endothelial, and immune cells, is vital. For this purpose, extensive use of intravital microscopy (IVM) could enhance the opportunity to characterize the TME on tissue and cellular scale to produce nanodrugs with foreseeable and desirable physicochemical and immunological behaviors [19].

IVM technique has been developed as an integration of methods including CLSM, multiphoton microscopy, and epifluorescence to dynamically evaluate tumor structure and physiological courses from tissue to subcellular level [149,150]. The IVM arrangements permit concurrent imaging of multiple TME constituents in combination with nanodrug circulation. IVM is a mature and powerful tool for illuminating biological events. Although label-free multiphoton IVM is striking for its nonperturbative character, its widespread uses have been mired mainly due to the inadequate contrast of each imaging technique and difficulties to combine them [150].

A simultaneous label-free auto fluorescence-multiharmonic (SLAM) microscopy has been developed to image generate from an extensive variety of cellular and extracellular components, including tumor cells, immune cells, vesicles, and tumor vessels. SLAM microscopy utilizes a single excitation source nonlinear imaging system that uses a custom-designed excitation source at 1110 nm and ultrafast pulse at 10 MHz to enable rapid, simultaneous, and effective acquisition of autofluorescence and SHG/THG from a wide array of cellular and extracellular machinery using 14 mW. The SLAM microscopy imaging technique shows versatility and efficiency for tracking cellular events as a label-free IVM modality. This imaging modality is used to perform nonperturbative intravital imaging of cellular dynamics and cell-stroma interactions. In addition to the intravascular motion tracking of leukocytes, leukocyte locomotion in the extravascular region was imaged with time-lapse imaging. In addition to detected leukocyte recruitment and clustering, their interaction with the collagen fibers, adipocytes, and blood vessels, was concurrently captured with the dynamic leukocyte behavior in a framework with the tumor or tissue microenvironment offering more insight into leukocyte behavior [150]. Thus, the utilization of innovative imaging modalities in combination is effective to visualize EPR-induced drug delivery processes across manifold spatial and temporal scales ranging from systemic biodistribution of nanodrugs to their delivery and significance upon distinct cellular levels.

## 6. Clinical Trials of Nanoparticles with EPR Enhancers and Their Clinical Translation

The field of nanomedicine is rapidly gaining interest due to its application in the diagnosis and treatment of diseases, especially the treatment of cancer. The promise of nanomedicine is in the tumor microenvironment. This microenvironment allows the nanoparticles to penetrate from the blood vessels into the interstitial space of the tumor and retain the particles in the tumor. This phenomenon, referred to as the enhanced permeability and retention effect (EPR), is used to deliver large drug loads or imaging agents to the tumor site better than small molecules. The enhanced permeability and retention (EPR) effect describes the preferential accumulation of nanoparticles in tumors due to their leaky vascular system and poor lymphatic drainage [151]. The EPR generated great enthusiasm for the development of nanoparticle therapy as an anticancer therapy. However, the results of preclinical studies assessing the EPR effect were inconclusive. Some reports confirm the preferential accumulation of nanodrugs in the tumor via EPR, while others show that the effect of EPR is strongly dependent on the tumor model. Today, the EPR remains a controversial topic in nanomedicine [152]. Hansen et al., using canine spontaneous tumors that closely mimic the human condition and 64Cu-labeled liposomes that can be imaged with positron emission tomography, showed that rapidly growing tumors such as carcinomas are highly vascularized and therefore have more porous and leaky blood vessels, while slow-growing tumors, such as sarcomas, are not very vascularized [153]. The main conclusion from this, and other EPR studies, is that the clinical application of EPR-enhancing agents, in combination with nanomedicine, requires preclinical studies conducted in appropriate models, the combination of nanomedicine with the EPR-enhancing strategy must be designed for a certain type of cancer, and side effects of EPR enhancing agents must be taken into consideration [154].

Many current cancer drug delivery systems attempt to go further than simply ensuring tumor accumulation. Attempts are being made to combine cancer cell-specific targeting with EPR-based accumulation. Intracellular delivery of drug-loaded macromolecule conjugates and pharmaceutical nanocarriers accumulated in tumors through the EPR effect can be facilitated in various ways [155].

One of the methods to facilitate the EPR effect is increasing the permeability of tumor vasculature by using vascular permeability factors, antiangiogenesis therapy, supplying oxygen to hypoxic regions, or thrombolysis therapy. Another option is to augment tumoral retention via ECM modulation. This strategy includes direct depletion of ECM components, inhibition of the synthesis of ECM components, or modulation of ECM-associated cells [156].

Optimization of nanomedicine design to enhance the EPR effect is also one of the strategies that are proposed, as well as a combination of the nanomedicine with the external stimuli, including ultrasounds, radiotherapy or hyperthermia or usage of nanomedicine modified with the cell-penetrating peptides [157]. Many attempts were made to improve the clinical translation of the EPR. The effort of studying the EPR effect paid off, as some of the developed nanomedicine-based EPR-enhancing strategies are currently undergoing clinical trials as shown in Table 3.

Despite the potential effectiveness of nanomedicine-based therapies in the treatment of cancer, patients’ response to such cancer treatments cannot be reliably predicted. However, the effect of EPR varies from patient to patient and may even vary within lesions in the same patient. Unlike all new cancer drugs, where protocols exist to distinguish between those who respond well to treatment and those who do not, nanomedicine-based cancer treatment technology does not have such a safety buffer. One of the proposed solutions is to perform noninvasive imaging of labeled nanocarriers that accumulate in tumors through the EPR effect. This makes it easy to identify low EPR patients who are unlikely to respond to treatment and refer them to established or experimental interventions. Similarly, patients with a high intensity of the EPR effect can count on relatively effective treatment with nanomedicine. The future for EPR-based nanomedicine is to create more personalized treatment strategies. [158,159].

## 7. Challenges of EPR-Based Drug Delivery into Solid Tumors

Nanoparticles pass through gaps between endothelial cells in tumor blood vessels, which are created during angiogenesis. These gaps have a size range of up to 2000 nm. Engineered NPs enter passively through the gaps and accumulate into tumors in an appropriate amount. However, after around three decades of limited clinical translation, the EPR mechanism is still facing difficulties. Chan W.C.W and coworkers performed a study analyzing the nanomedicine delivery literature from the year 2005 to 2015 and exhibited that only 0.7% NPs that were administered systemically reach tumors [160]. In absolute terms, this number is small and challenging to the efficiency of EPR-based drug delivery. However, in relative terms, a delivery efficiency of 0.7% is significantly higher than that of chemotherapeutics being utilized in clinics such as doxorubicin, paclitaxel, and docetaxel. Several studies have demonstrated that only 0.1 to 0.2% of the injected dose is efficient against tumor cells and exhibits significant anticancer effects [20,123,161].

To overcome the challenges of EPR-based drug delivery and enhance NP-based anticancer therapy, further multimodal studies of the drug delivery mechanisms are essential. A recent study by Chan W.C.W et al. demonstrated that inter-endothelial gaps in tumors may not be responsible for the transport of nanodrugs into solid tumors [161]. They showed around 97% of the NPs are delivered to tumors by endothelial cells through an active process of transcytosis instead of passive transport. Transcytosis is a metabolically active process that necessities endothelial cells to reorganize their cytoskeleton and cell membrane, involving vesicle formation transporting NPs through the cytoplasm [123,162]. Chan W.C.W et al. performed these multimodal studies utilizing mouse models, TEM studies, imaging techniques, etc. Contrary to the EPR mechanism, the frequency of the gaps on the endothelial lining in tumors is too low to account for the NP accumulation in the tumor site, which was observed by TEM analysis [161]. This study’s outcome is a challenge for the long-established EPR mechanism of drug delivery to solid tumors. The study may stimulate researchers to develop techniques that involve the active transcytosis process to enhance nanodrug delivery efficiency in addition to a passive EPR-based drug delivery mechanism. The active versus passive drug delivery controversy may be resolved through multidisciplinary research efforts by life scientists, oncologists, and nanomaterial scientists. Therefore, defining the dominant mechanism of the entry of NPs into tumors may help engineer effective delivery carriers to enhance cancer therapy [161].

## 8. Conclusions and Future Perspective

Targeting the dynamics of the EPR effect and enhancement of the therapeutic effects of nanodrugs through EPR enhancers is a vibrant strategy to progress cancer therapy. The scarcity of data on the effectiveness of EPR in humans hinders the clinical advancement of cancer drugs. Molecular targeting, physical modification, or physiological reformation of the TME may be considered significant strategies for enlightening the EPR effect. The utilization of nanodrugs that can target TME or TME constituents is one of the best effective ways to improve the targeting ability of nanodrugs for overcoming tumor heterogeneity and enhancing the EPR effect. Approaches that enhance the endothelial pores which assist in the access of nanodrugs to extravasate into TME by using NO, nitroglycerin, TNF-α, angiotensin, etc. have the potential for EPR enhancement and advancing the therapeutic effect.

Innovative imaging modalities for picturing EPR-prompted nanodrug delivery in tumors and the use of improved animal models are obligatory to augment the EPR effect. Noninvasive imaging of labeled nanocarriers that aggregate in tumors through the EPR effect may make it easy to recognize low EPR patients who are not likely to respond to therapy and refer them to established or experimental interventions. Patients with a high intensity of the EPR effect can count on comparatively effective therapy with nanodrugs. This approach may help shape the future for EPR-based nanomedicine to predict more personalized treatment strategies.

Integration of nanodrugs with imaging modalities is vital for visualizing tumor-targeted drug delivery progressions, and inter- and intraindividual heterogeneity in EPR-based drug delivery. Determinants of the EPR effect and nanodrug action happen across manifold spatial and temporal scales ranging from systemic biodistribution of nanodrugs to their release and significance upon discrete cellular levels. Therefore, the utilization of innovative imaging modalities in combination is effective to visualize EPR-induced drug delivery processes from systemic biodistribution of nanodrugs to their delivery on a cellular level and enhances the EPR-based therapeutic effect. Finally, identifying the dominant mechanism of entry of NPs into tumors, whether it is an active transcytosis or passive process, or both, may be supportive in engineering innovative delivery carriers to enhance cancer therapy.

## Figures and Tables

**Table 1:** Types of vessels in solid tumors [15].

Types of Vessels	Major Characteristics
Mother vessels	Large in size with thin walls and good permeability
Glomeruloid microvascular	Poorly organized cells of proliferation (Endothelial cells, pericytes, basement membrane)
Capillaries	Include primary and glomeruloid microvascular vessels
Vascular malformations	Irregular coverage of smooth muscle tissues
Feeding arteries	Large vessels having complete structures of capillaries
Drainage veins	Very large vessels

**Table 2:** Types of EPR-effect enhancers and mechanism of action [44].

Novel EPR Effect Enhancers	Mechanism of Action	References
Isosorbide dinitrate and Sildenafil	The therapeutic efficacy of the drug was increased 2–4 fold after blood flow restoration through the generation of NO	[9]
Nitroglycerine, hydroxyurea, and L-arginine	Generation of NO resulted in 1.5–2 times improved delivery and 2–4 fold antitumoreffects of model drugs	[108]
Styrene maleic acid copolymer and PEG-hemin	1.5–2 times greater tumor delivery of nanodrug when combined with generators of CO	[109]
Human serum albumin nanoparticles containing CO donor and photosensitizer (MnCO and IR780)	Combined CO gas therapy and phototherapy significantly inhibited the growth of tumors through synergism	[110]
Losartan	Angiotensin II receptor antagonist with marked fibrinolytic activity inhibited collagen production and enhanced penetration.	[111,112]

**Table 3:** Example of clinical trials of combination of nanomedicine with pharmacologically-based and physically-based EPR enhancing strategies. Adapted from [23]. Copyright (2023), with permission from Elsevier.

EPR Enhancer	Targeted Drug	Carrier	Identifier	Clinical Phase	Strategy
PHYSIACL STRATEGIES					
	TermoDOX®	Liposomes	NCT04791228	II	Hyperthermia
	Nab-paclitaxel	Protein based nanoparticles	NCT01847326	I	Radiotherapy
	Nab-paclitaxel, nivolumab	Protein based nanoparticles	NCT03107182	II	Radiotherapy
	Irinotecan, PD-1 antibody	Liposomes	NCT04569916	II	Radiotherapy
AGUIX®	AGuIX®	Nanoparticles	NCT04881032	I/II	Radiotherapy
NBTXR3	NBTXR3, cetuximab	Nanoparticles	NCT04892173	III	Radiotherapy
DEFINITY		Lipid microspheres	NCT02764801	III	Ultrasound
MICROBUBBLE	Nab-paclitaxel	Protein based nanoparticles	NCT04528680	I/II	Ultrasound
SONAZOID		Microbubbles	NCT05105087	I	Ultrasound
PHARMACOLOGICAL STRATEGIES					
BEVACIZUMAB AND ITS BIOSIMILAR	Nab-paclitaxel, avelumab, ETBX-011, GI-4000	Nanoparticles	NCT03136406	I/II	Vascular normalizer
BEVACIZUMAB AND ITS BIOSIMILAR	Doxorubicin	Liposomes	NCT01802749	III	Vascular normalizer
BEVACIZUMAB AND ITS BIOSIMILAR	Atezolizumab, platinum	Liposomes	NCT02891824	III	Vascular normalizer
BEVACIZUMAB AND ITS BIOSIMILAR	Paclitaxel conjugated with the albumin	Protein based nanoparticles	NCT00404404	II	Vascular normalizer
BEVACIZUMAB AND ITS BIOSIMILAR	Rapamycin conjugated with the albumin	Protein based nanoparticles	NCT03463265	II	Vascular normalizer
HYDROXYUREA	Nab-paclitaxel	Protein based nanoparticles	NCT01847326	I	Vascular mediation
HYDROXYUREA	Nab-paclitaxel	Protein based nanoparticles	NCT02258659	II	Vascular mediation
CEND-1	Nab-paclitaxel and gemcitabine	Protein based nanoparticles	NCT05052567	II	Tumor penetrating peptide
CEND-1	Nab-paclitaxel and gemcitabine	Protein based nanoparticles	NCT05042128	II	Tumor penetrating peptide

## Data Availability

No supplementary data is available.
